# Supplementary material for: Co-translational assembly of mammalian nuclear multisubunit complexes
Source: Nat Commun. 2019 Apr 15;10:1740. doi: 10.1038/s41467-019-09749-y (PMC6465333; doi:10.1038/s41467-019-09749-y)
Supplement: Supplementary file 1 — Supplementary Information [file 41467_2019_9749_MOESM1_ESM.pdf]

Supplementary Information (Figures and Tables)

Co-translational assembly of mammalian nuclear multisubunit complexes

Ivanka Kamenova[1,2,3,4,#], **Pooja Mukherjee**[1,2,3,4,#], **Sascha Conic**[1,2,3,4,], **Florian Mueller**[5], **Farrah El-Saafin**[1,2,3,4,], **Paul Bardot**[1,2,3,4,], **Jean-Marie Garnier**[1,2,3,4], **Doulaye Dembele**[1,2,3,4,], **Simona Capponi**[6], **H.T. Marc Timmers**[6], **Stéphane D. Vincent**[1,2,3,4,] **and László Tora**[1,2,3,4, *]

[1]Institut de Génétique et de Biologie Moléculaire et Cellulaire, Illkirch, France;

[2]Centre National de la Recherche Scientifique, UMR7104, Illkirch, France;

[3]Institut National de la Santé et de la Recherche Médicale, U1258, Illkirch, France;

[4]Université de Strasbourg, Illkirch, France;

[5]Computational Imaging & Modeling Unit, Institut Pasteur, Département Biologie Cellulaire et Infections, 25-28 rue du Docteur Roux, 75015 Paris, France;

[6]German Cancer Consortium (DKTK) partner site Freiburg, German Cancer Research, Center (DKFZ) and Department of Urology, Medical Center-University of Freiburg, Germany.

[#]These authors contributed equally

[*]Correspondence should be addressed to L.T. laszlo@igbmc.fr

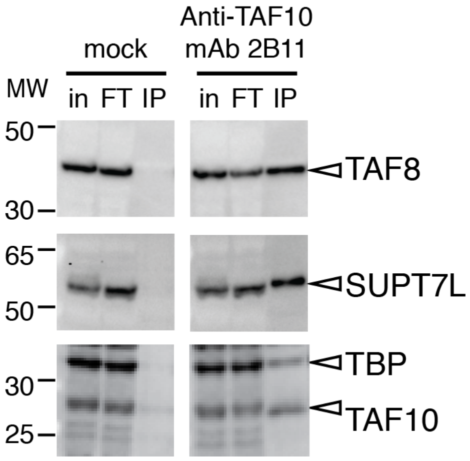

Supplementary Figure 1

Western blot analysis of immunoprecipitations from E14 mESC whole cell extract using mock (anti-GST) or anti-TAF10 (6TA 2B11) antibodies. 6TA 2B11 recognizes the N-terminal unstructured region of TAF10. Blots were probed with antibodies against the indicated proteins. In = input, FT = flow-through, IP = eluate from IP. Molecular weight (MW) markers are shown in kDa. Source data provided as a Source Data File.

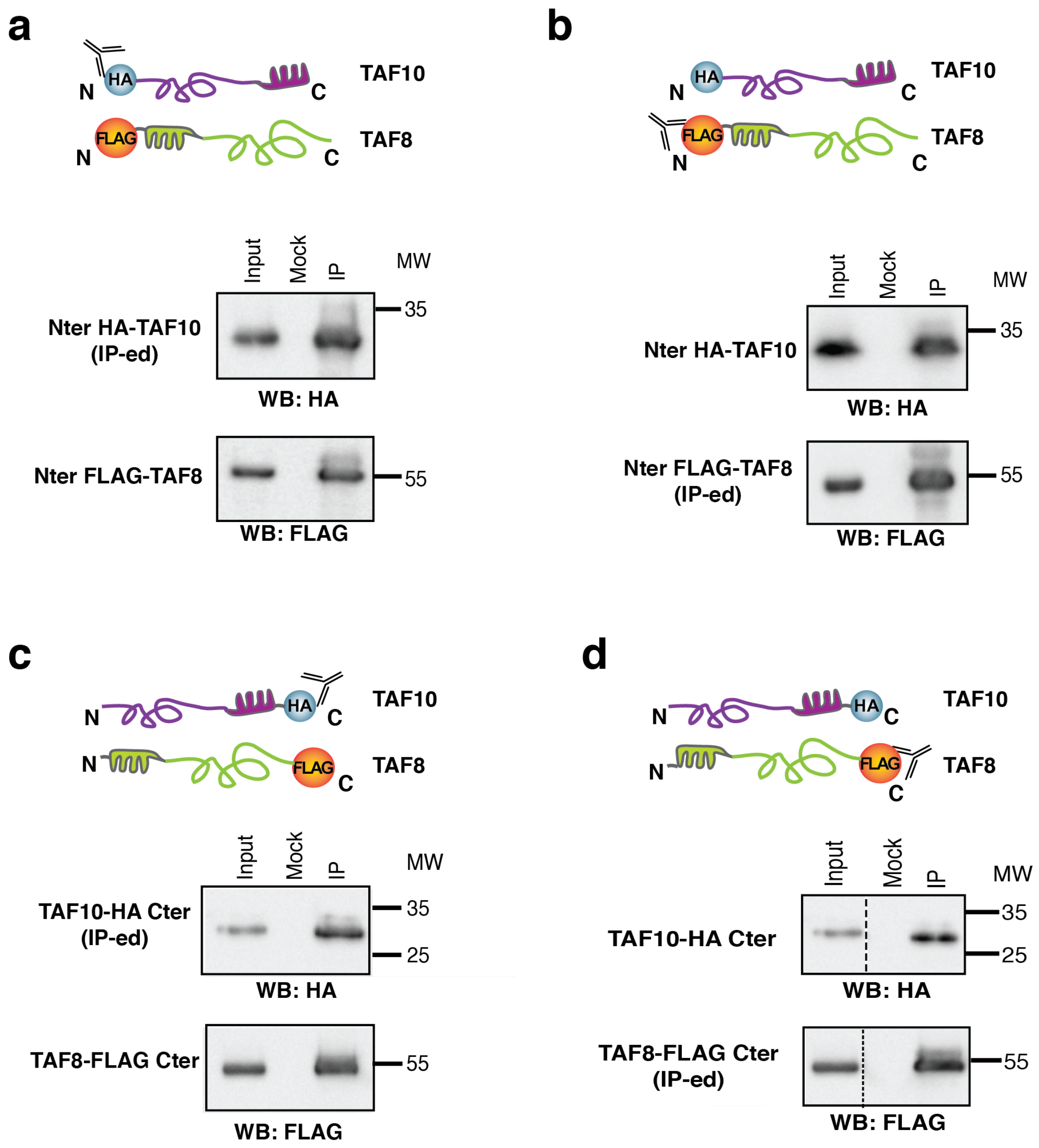

Supplementary Figure 2

(a-d) Western blot analyses of RIPs analysed in Fig. 2. HeLa cells transfected with expression constructs for N terminal (**a-b**) or C terminal (**c-d**) tagged HA-TAF10 and FLAG-TAF8 proteins. Blots were probed with anti-FLAG M2 and anti-HA antibodies as indicated. Molecular weight (MW) markers are shown in kDa. In = input, FT = flow-through, IP = eluate from IP. Molecular weight (MW) markers are shown in kDa. Source data provided as a Source Data File.

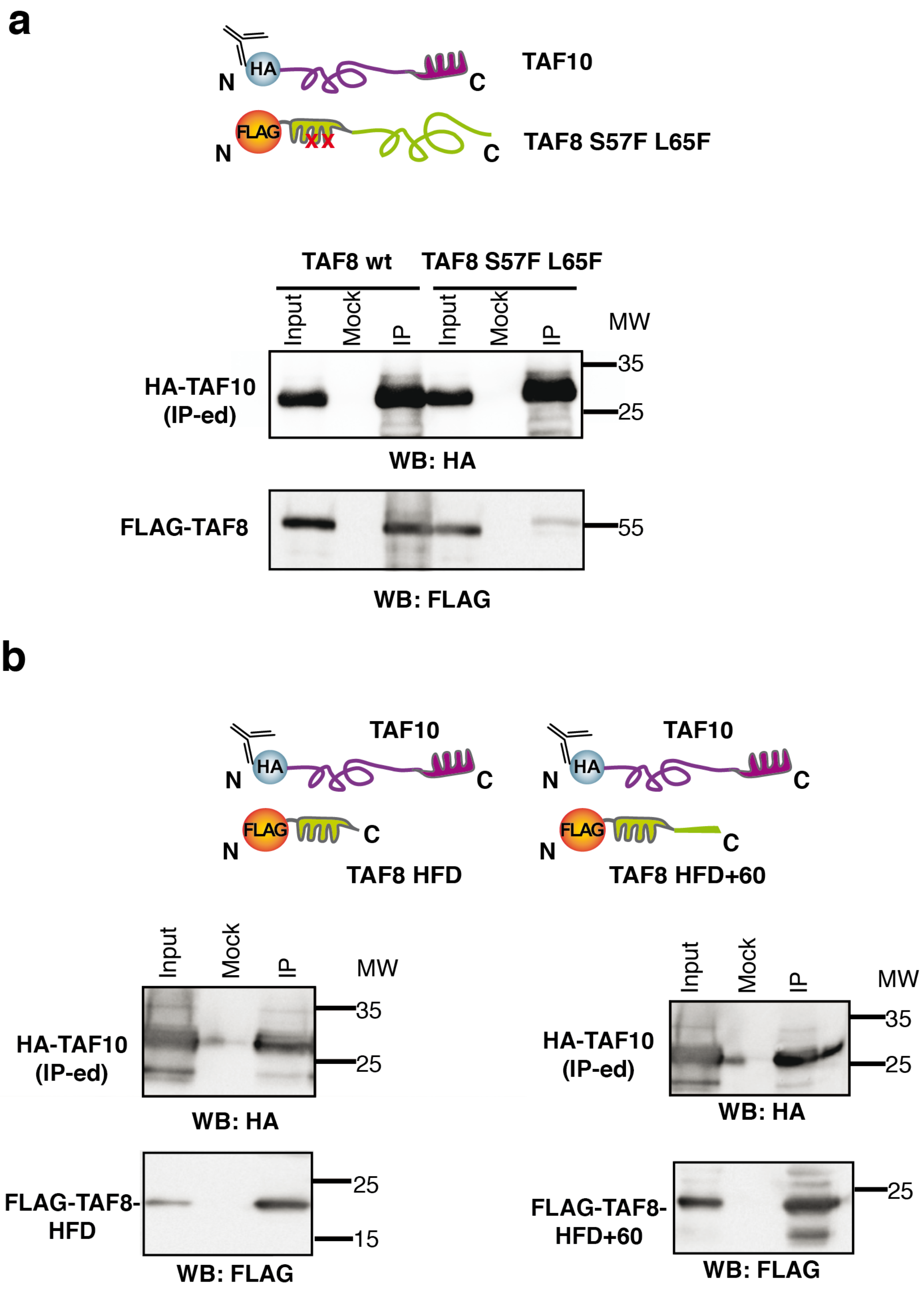

Supplementary Figure 3

Western blot analyses of RIPs analysed in Fig. 3. Blots were probed with anti-HA and anti-FLAG M2 antibodies as indicated. Hela cells transfected with expression constructs coding for HA-TAF10 and mutant FLAG-TAF8 S57F L65F proteins (**a**) and coding for HA-TAF10 and minimal TAF8 HFD (left) or TAF8 HFD extended with 60 amino acids (TAF8 HFD+60, right) (**b**). Molecular weight (MW) markers are shown in kDa. In = input, FT = flow-through, IP = eluate from IP. Molecular weight (MW) markers are shown in kDa. Source data provided as a Source Data File.

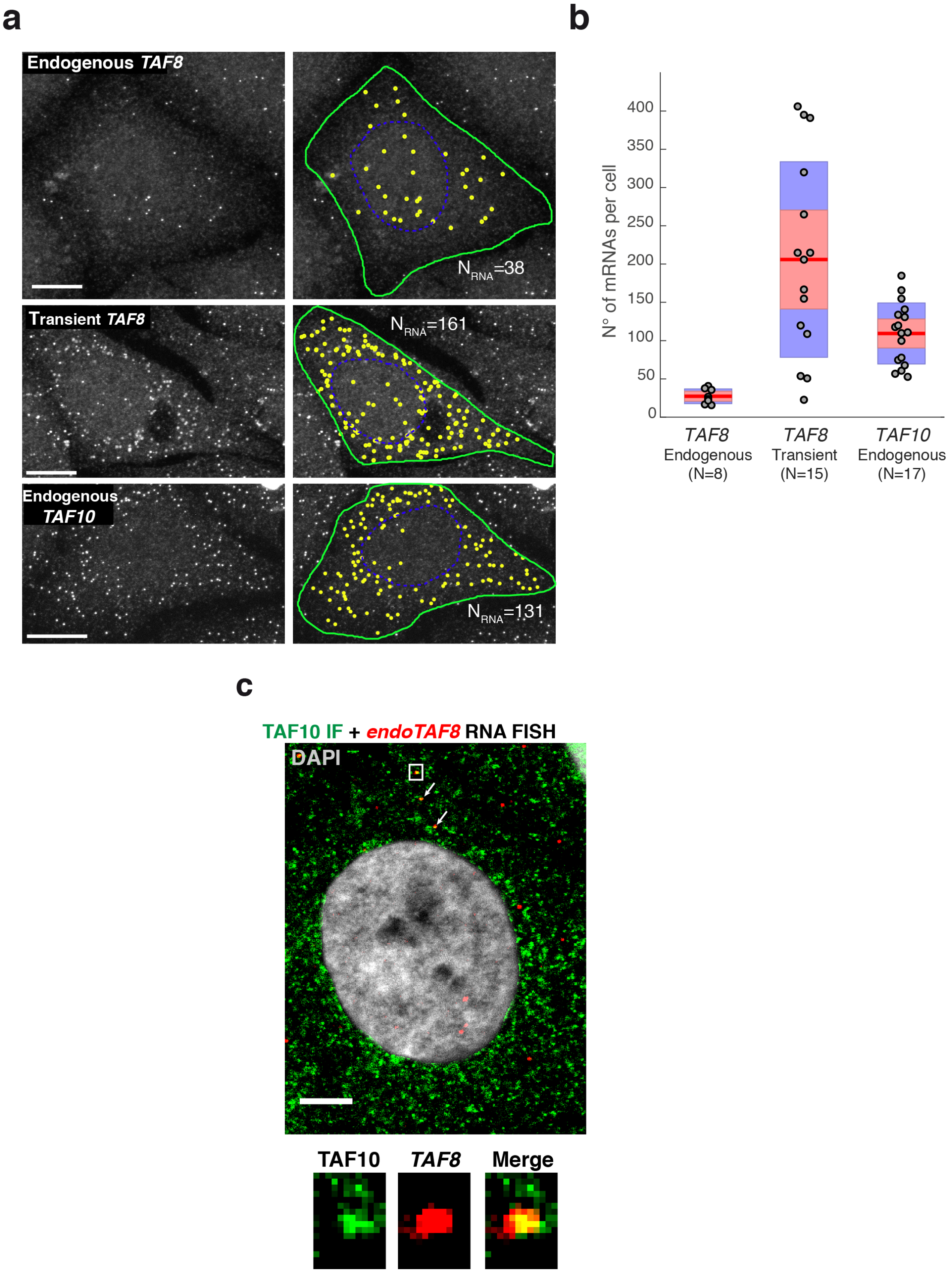

Supplementary Figure 4

(**a**) Representative single colour smiFISH images with Cy3-labelled probes recognizing the indicated mRNAs. Z projections of confocal images are shown. On the right, the cell boundaries are shown in green and the nuclei in blue. Scale bars are 10 µm in each case. RNAs were detected with FISH-quant. N indicates the number of mRNA molecules in the cytoplasm of each individual cell. **(b)** Boxplot showing the comparison of the number (N°) of mRNAs under the conditions tested (as indicated). Each grey dot represents one cell. Red horizontal lines are mean values, 95% confidence interval is shown in pink, and standard deviation in blue. N = number of cells counted. (**c**) IF-smiFISH images of HeLa cells expressing either endogenous (endo) *TAF8* mRNA or TAF10 protein. Labels: red, Cy3-labelled *TAF8* probes; green, Alexa-488 labelled secondary antibody for TAF10 protein; co-localizing spots are indicated with white arrows. A typical cell counterstained with DAPI (grey) is shown. The nuclear signal in the green channel (TAF10 IF) was removed by masking the nucleus and using the "clear" option in ImageJ. Zoom-in regions shown are indicated with a white rectangle. Scale bar (5 µm).

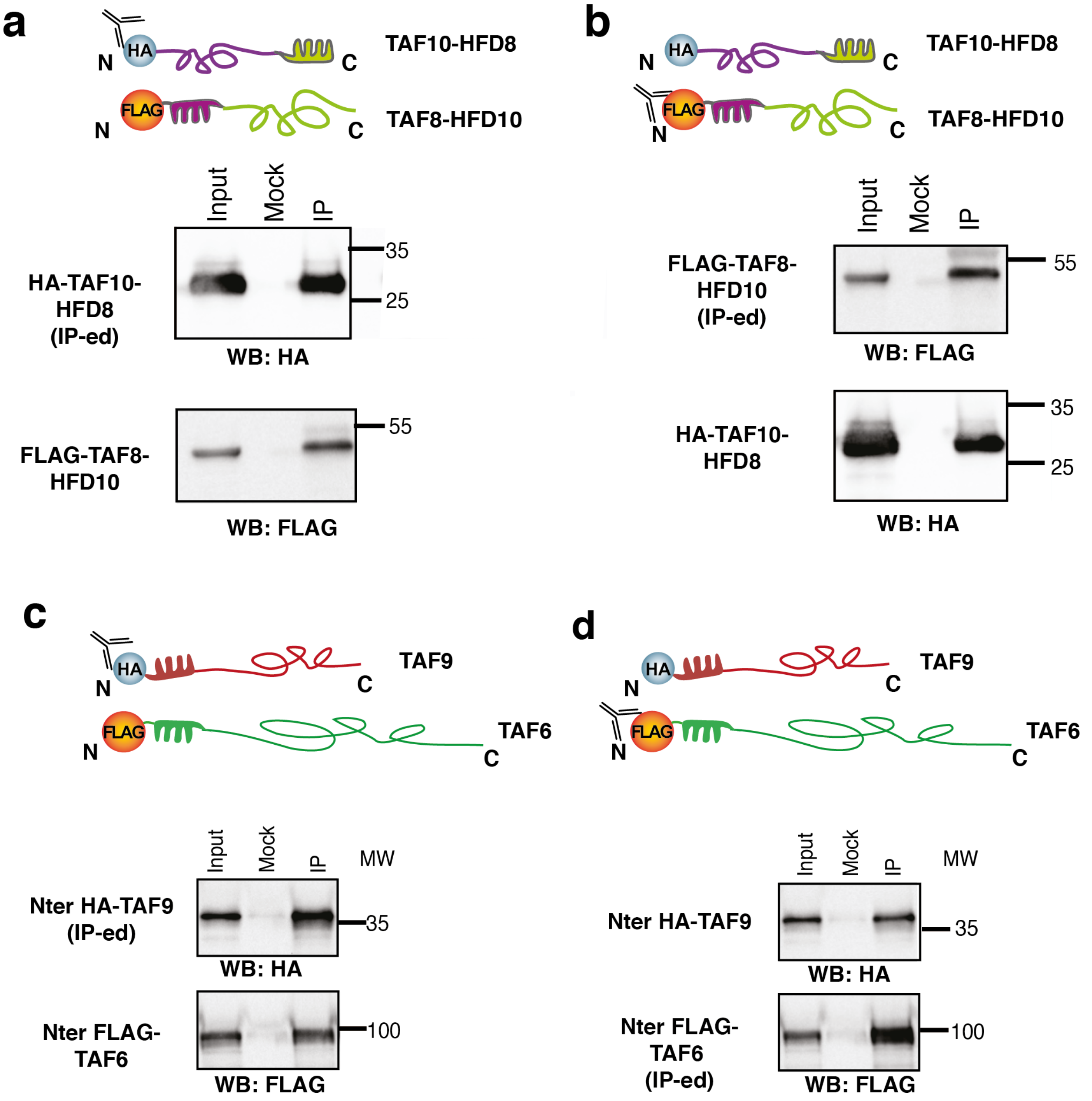

Supplementary Figure 5

Western blot analyses of RIPs from polysome extracts corresponding to Fig. 6 (**a-d**). HeLa cells transfected with expression constructs coding for HFD domain-swapped TAF10 and TAF8 (**a-b**) and with expression constructs coding for TAF6 and TAF9 (**c-d**). Blots were probed with anti-FLAG M2 and anti-HA antibodies as indicated. Molecular weight (MW) markers are shown in kDa. In = input, FT = flow-through, IP = eluate from IP. Source data provided as a Source Data File.

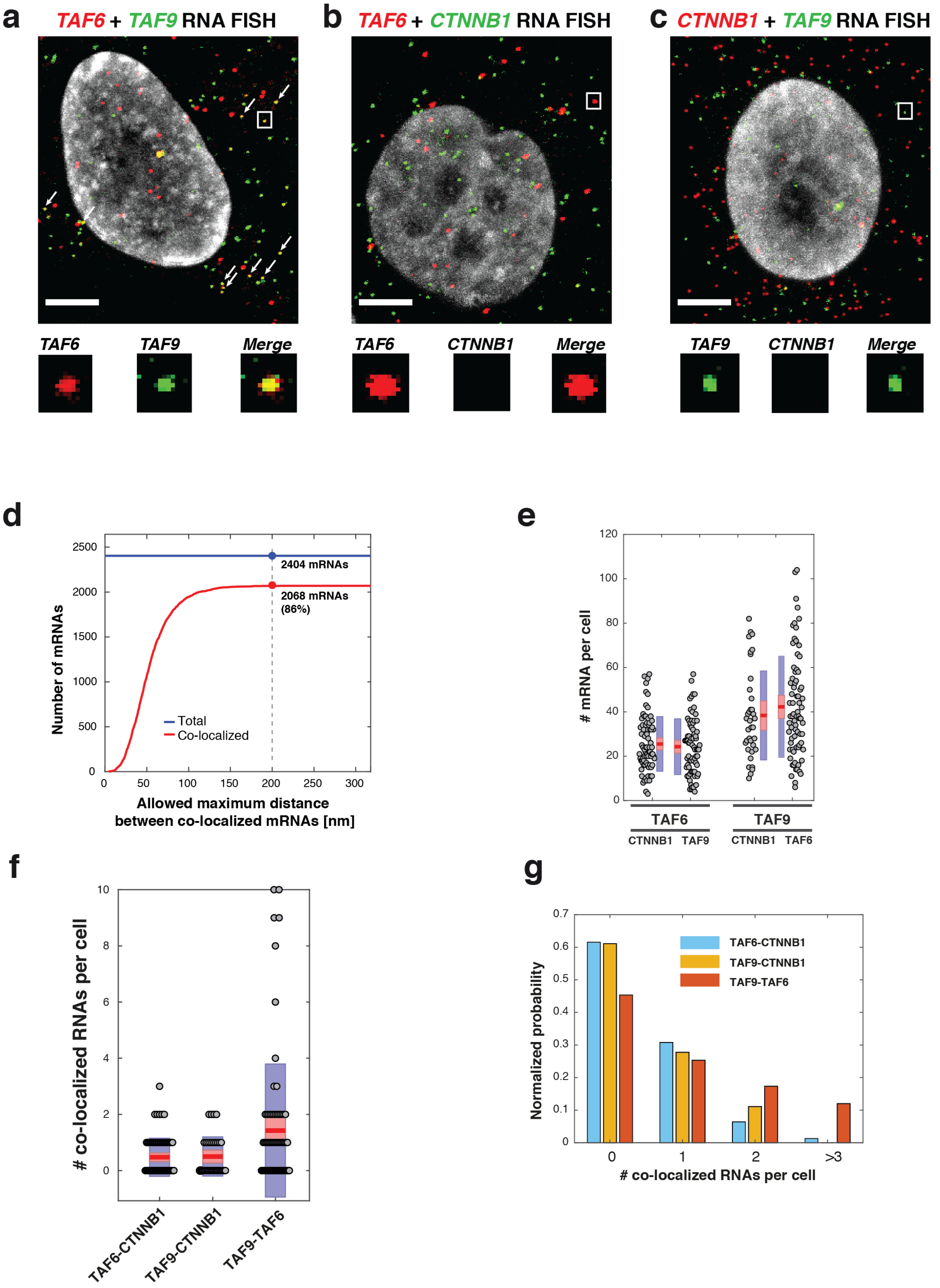

Supplementary Figure 6

Representative dual colour smiFISH images with (**a**) Cy3-labelled *TAF6* and digoxigenin (DIG)-labelled *TAF9* probes, (**b**) Cy3-labelled *TAF6* and DIG-labelled *CTNNB1* probes, (**c**) Cy3-labelled *CTNNB1* and DIG-labelled TAF9 probes. The DIG labelled probes were then detected with fluorescein-labelled anti-digoxigenin antibodies. Co-localizing spots are indicated with white arrows.

A typical cell recorded in each case and after counterstaining the nucleus with DAPI (grey) is shown. The nuclear signal was masked using the "clear" option in ImageJ. Zoom-in regions shown under every image are indicated with a white rectangle. Scale bar: 5 µm. (**d**) Analysis to determine allowed distance threshold for two RNAs to be considered co-localized. Probe-pool for *TAF9* was split in two and labelled with two different colours. Co-localization between these two channels was determined for different distance threshold. Co-localization reaches a plateau at around 200 nm, with co-localization percentage of 85%. Both, distance threshold and co-localization percentage are in similar to earlier studies (Tsanov et al. 2016). (**e**) Comparison of RNA expression levels per cell for TAF6 and TAF9 in either the respective negative control experiments (against *CTNNB1*), or the *TAF6-TAF9* co-localization experiment. Plot shows that detected expression levels are similar. (**f**) Number of co-localized RNAs per cell. Shows significant increase of co-localization in *TAF6-TAF9* experiments compared to negative controls. In panels (e-f), each grey dot represents one cell. Red horizontal lines are mean values, 95% confidence interval is shown in pink, and standard deviation in blue. (**g**) Same data as in (f) but shown as a histogram. Only in the *TAF6-TAF9* experiments a substantial number of cells have more than 2 co-localized RNAs per cell.

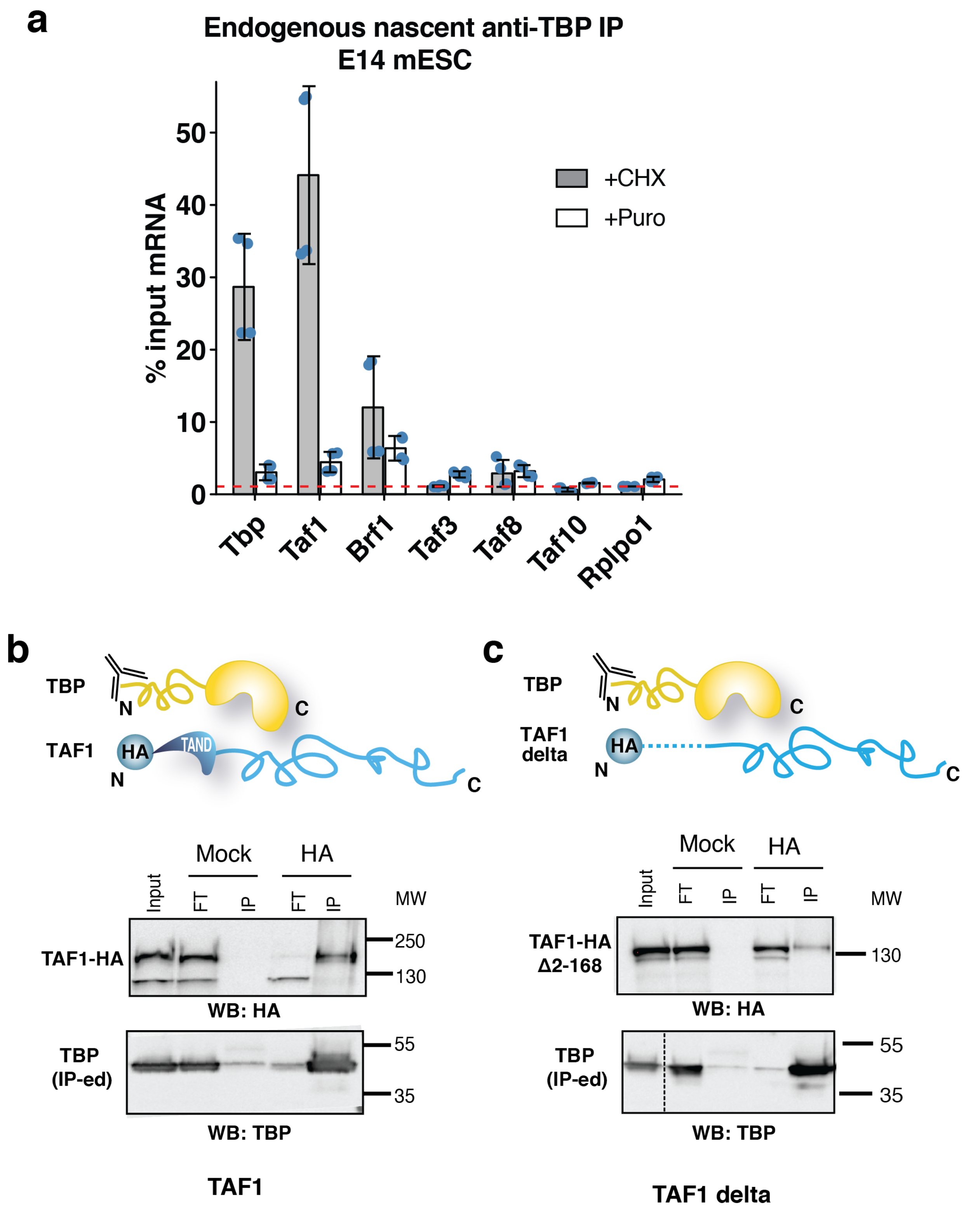

Supplementary Figure 7

(a) RIP-qPCR using TBP antibody from mESC polysome extracts. Values are expressed mean ±S.D. from 2 biological replicates and two technical replicates (represented by blue dots). **(b-c)** Western blot analyses of RIPs with TBP antibody from polysome extracts corresponding to Fig. 7(**c-d**). **(b)** HeLa cells transfected with expression constructs coding for TBP and wild type HA-TAF1, **(c)** HeLa cells transfected with expression constructs coding for TBP and HA-TAF1 with N-terminal deletion of the first 168 amino acids. Molecular weight (MW) markers are shown in kDa. In = input, FT = flow-through, IP = eluate from IP. Source data provided as a Source Data File.

Supplementary Tables

Supplementary Table 1: primers used in this study

**gene**	**organism**	**name**	**sequence**
TBP	human	hTBP1-F<br>hTBP1-R	TCATACCGTGCTGCTATCT<br>CTCCCTCAAACCAACTTGTC
TAF1	human	hTAF1-F<br>hTAF1-R	TTTGTACCTGCCTTGTTCC<br>GCCCATCTTTCAGTCTCATC
TAF2	human	hTAF2-F<br>hTAF2-R	CATGTGTACCGCCAAAGT<br>GCAGTTGCTTCTGTGTAAATC
TAF3	human	hTAF3-2F<br>hTAF3-2R	GACGACTGCGATGACTGGTA<br>CTTCTTGTTCGCACACTTGG
TAF5	human	hTAF5_F_558<br>hTAF5_R_627	AGTTGGAAGTGTTGCTGTGG<br>TCCTTGTTGGTTGTAGGCTGAC
TAF6	human	hTAF6_264_352_F<br>hTAF6_264_352_R	CCAGGAGTTCATTCCTTTCC<br>TGATGTCGCTCAGATCAACC
TAF7	human	hTAF7_F_76<br>hTAF7_R_164	TCTACTGTGAGAAGGGCAGTAC<br>ATTCCATGACGCCCATCAGG
TAF8	human	hTAF8-3F<br>hTAF8-3R	ACAGAGGCAGGGTTTGAGAGT<br>AGACTTGGCACTTCTCCCAAT
TAF9	human	hTAF9_F<br>hTAF9_R	GGAGTTTGCCTTCCGATATG<br>CGCACATCATCTGCATCAAC
TAF10	human/mouse	TAF10s<br>TAF10as	TGCCAATGATGCCCTACAGC<br>AGGGCAGGGGTCAAGTCCTC
SPT7L	human	hSPT7L-3F<br>hSPT7L-3R	AGAATCCCAATGCACCATTC<br>GCCAGCTGAGTTCAGTCACA
BRF1	human	BRF1-2F<br>BRF1-2R	GAGGTGCAGTTCGTGGAGAG<br>CTCTCGACTCCTTCCCCAGA
BTAF1	human	BTAF1-1F<br>BTAF1-1R	CCCTCAGGTCCAACAATGCA<br>GGCCAGACACGTGGAACTAA
*TAF8*_deletion	human	FLAG_F<br>TAF8_HFD_R	GACATCGATTACAAGGATG<br>AGGGTTAGTGGACTGTTTA
*TAF1*_deletion	human	TAF1_HA_F<br>TAF1_HA_R	CTACGACGTGCCCGACTAC<br>GGCAATGATGGAGGGCAAGA
PPIB	human	Cyclopillin B s<br>Cyclopillin B as	CCGAACGCAACATGAAGGTG<br>ACCAAAGATCACCCGGCCTA
GAPDH	human	GAPDH-2F<br>GAPDH-2R	TCGACAGTCAGCCGCATCTTCTTT<br>ACCAAATCCGTTGACTCCGACCTT
ENY2	human	ENY2_fw<br>ENY2_rev	GGAGAAAGAGAACGCCTCAAA<br>AGTGATTTCAGCCACCAAGTCA
GANP	human	GANP_fw<br>GANP_rev	CACGAGCCAGCAGCAGAAGTTC<br>CATCCTGTATCGTCCGACCA
CETN2	human	CENTR2_fw<br>CENTR2_rev	GGACAGGAAAAATGAACTTTGGTGA<br>GGCCACGCGTTTCAGATTTT
CETN3	human	CENTR3_fw	AGAGCCTTGGGGTTTCATGTAA

		CENTR3_rev	TTCTTCATGGGGATCTCTCTTTCC
ATXN7L3	human	ATXN7L3_fw<br>ATXN7L3_rev	CTGGGAATGGGTCGGAACAG<br>CCGAGCCATAGGACCAGTCG
ATXN7	human	ATXN7_fw<br>ATXN7_rev	GCGAAGTCATGGGGCTCTGT<br>TTGAAATGCCTGCGGTTTGA
USP22	human	USP22_fw<br>USP22_rev	TTGCAGATGCCTTTCTGTTG<br>TAGAAAACCGCGAGATGCTT
Tbp	mouse	TBP_mouse_F1<br>TBP_mouse_R1	AGCAACAAAGACAGCAGCAG<br>CTGTGTGGGTTGCTGAGATG
Taf1	mouse	TAF1_mouse_F1<br>TAF1_mouse_R1	TGGAGATGGTGATCTTGCAG<br>TCCTCATCATCTTCGCCTTC
Taf3	mouse	TAF3_mouse_F1<br>TAF3_mouse_R1	TGCTGGCTCCATTTGCAAAG<br>TTTTCTGACCTGGAGAGCTAGC
Taf8	mouse	TAF8_mouse_F1<br>TAF8_mouse_R1	ATATCAGCACGGACGATTCC<br>GGTTATCGATGACGCTCTCC
Taf10	human/mouse	TAF10s<br>TAF10as	TGCCAATGATGCCCTACAGC<br>AGGGCAGGGGTCAAGTCCTC
Spt7l	mouse	Spt7 NMD F<br>Spt7 NMD R	GGAGCATTGGGATTTTTACAGT<br>TGTGAAGGCTGAAGAGAGTGAA
Brf1	mouse	BRF1_mouse_F1<br>BRF1_mouse_R1	AGTATCCATGACAGCCTTGAGG<br>TGCAACCAAAAGTGCTGCTC
Rplp0[1]	mouse	RPLP0_mouse_F1<br>RPLP0_mouse_R1	TTCTGAGTGATGTGCAGCTG<br>GGAGATGTTCAGCATGTTCAGC
Gapdh	mouse	mGapdh_F<br>mGapdh_R	TTCACCACCATGGAGAAGGC<br>CCCTTTTGGCTCCACCCT
Taf8_2	mouse	mTaf8_F<br>mTaf8_R	GAGCTCCTTGCTGACAGAGG<br>GCACTTCTCCCGATTTCTGA
Taf10_2[1]	mouse	mTaf10_F<br>mTaf10_R	CCACGCATAATTCGGCTCAT<br>CCTCCATGGTTAGGTGTACT

Supplementary Table 2: antibodies used in this study

Name	Type	Antigen	Purpose	Dilution	Source
23TA-1H8	mouse monoclonal, ascites	hTAF10 aa 1-20	RIP (human)	2 µl ascites for 20 µl Protein G Dynabeads	Wieczorek *et al*, 1998[2]
6TA-2B11	mouse monoclonal, ascites	hTAF10 aa 89-100	RIP (mouse), protein IP, western blot	2 µl ascites for 20 µl Protein G Dynabeads (RIP, protein IP) 1:1000 (WB)	Mohan *et al*, MCB, 2003[3]
3TF1-3G3	mouse monoclonal, ascites	hTBP aa 1-18	RIP (human, mouse), western blot	2 µl ascites for 20 µl Protein G Dynabeads (RIP) 1:1000 (WB)	Brou *et al*, EMBO J, 1993[4]
3F10	rat monoclonal	HA	western blot	1:500	Roche (Sigma) cat #: 11867423001
anti-FLAG M2	mouse monoclonal, affinity purified	FLAG	western blot	1:2000	Sigma, F1804
#2440	rabbit polyclonal, antigen affinity purified	hTAF1	western blot	1:1000	Choukrallah *et al*, 2011[5]
#2325	rabbit polyclonal, antigen affinity purified	ATXN7L3	western blot	1:1000	Zhao *et al* 2008[6]
ab113295	rabbit polyclonal	hGANP	western blot	1:500	Abcam
SUPT7L	rabbit polyclonal, antigen affinity purified	SUPT7L	western blot	1:5000	Bethyl, A302-803A
#3478	rabbit polyclonal, antigen affinity purified	TAF8	western blot	1:1000	Bardot *et al*, 2017[1]
15-TF2-1D10	mouse monoclonal, ascites	GST	RIP (human, mouse), protein IP	2 µl ascites for 20 µl Protein G Dynabeads	Nagy *et al*., 2010[7]
TUBULIN	mouse monoclonal	TUBULIN	western blot	1:20000	SIGMA-T6557

Supplementary Table 3: plasmids used in this study

Plasmid	*Description*	*Source*
pXJ41-TAF10-Nter-2HA	Eukaryotic expression plasmid containing 2HA-hTAF10 for N-terminal tagging of hTAF10	Jacq et al,1994[8]
pXJ41-TAF8-Nter-3FLAG	Eukaryotic expression plasmid containing 3FLAG-hTAF8 for N-terminal tagging of hTAF8	This study
pXJ41-TAF10-Cter-2HA	Eukaryotic expression plasmid containing 2HA-hTAF10 for C-terminal tagging of hTAF10	This study
pXJ41-TAF8-Cter-3FLAG	Eukaryotic expression plasmid containing 3FLAG-hTAF8 for C-terminal tagging of hTAF8	This study
pXJ41-TAF9-Nter-2HA	Eukaryotic expression plasmid containing 2HA-hTAF9 for N-terminal tagging of hTAF9	This study
pXJ41-TAF6-Nter-3FLAG	Eukaryotic expression plasmid containing 3FLAG-hTAF6 for N-terminal tagging of hTAF6	This study
pXJ41-TAF8-L65F-S57F-Nter-2HA	Eukaryotic expression plasmid containing N-terminally 3FLAG tagged hTAF8 with two point mutations L65F S57F	This study
pXJ41-TAF8HFD-Nter-3FLAG	Eukaryotic expression plasmid containing N-terminally 3FLAG tagged histone fold domain of hTAF8	This study
pXJ41-TAF8HFD60aa-Nter-3FLAG	Eukaryotic expression plasmid containing N-terminally 3FLAG tagged hTAF8 histone fold domain with its 60 aa extension	This study
pXJ41-TAF10(HFD)TAF8-Nter-2HA	Eukaryotic expression plasmid containing N-terminally 2HA tagged hTAF10 with the histone fold domain replaced with that of hTAF8	This study
pXJ41-TAF8(HFD)TAF10-Nter-3FLAG	Eukaryotic expression plasmid containing N-terminally 3FLAG tagged hTAF8 with the histone fold domain replaced with that of hTAF10	This study
pXJ41-TBP	Eukaryotic expression plasmid containing hTBP	May et al,1996[9]
pXJ41-TAF1-Nter-HA	Eukaryotic expression plasmid containing	This study

	1HA-hTAF1 for N-terminal tagging of hTAF1	
pXJ41-ΔTAF1-Nter-HA	Eukaryotic expression plasmid containing N-terminally tagged 1HA-hTAF1, amino acid 2-168 deleted	This study

Supplementary Table 4: smiFISH probes

TAF8 probes

**Name**	**Probe Sequence (including FLAPY Sequence)**
hTAF8_YDG30_001	TCAGGAGAGGGACTTCTTCCTGCGGATTACACTCGGACCTCGTC GACATGCATT
hTAF8_YDG30_002	TGCAGGACAGAGGTGTTCTCCTTCTCTTACACTCGGACCTCGTCG ACATGCATT
hTAF8_YDG30_003	CTCCAGAATCCTCCATGCTGATATGATTACACTCGGACCTCGTCG ACATGCATT
hTAF8_YDG30_004	AGCAAGGTTCTCTGTGTCTGTCTGTTCATTACACTCGGACCTCGT CGACATGCATT
hTAF8_YDG30_005	CTGCTCCGAGGAATCTGTCTCTTCCATTACACTCGGACCTCGTCG ACATGCATT
hTAF8_YDG30_006	TGTTGCATCTCCAGTTCAGACGGAAGAAGTTACACTCGGACCTC GTCGACATGCATT
hTAF8_YDG30_007	CTGTCAGGTAGGGGATGGTGAAAGGTCTGTTACACTCGGACCTC GTCGACATGCATT
hTAF8_YDG30_008	AGCAATCAATGGAAATGTGCTGACGTCATTTACACTCGGACCTC GTCGACATGCATT
hTAF8_YDG30_009	TTGGGCTTCTTCACCGGCCGCAGATATTACACTCGGACCTCGTCG ACATGCATT
hTAF8_YDG30_010	TTGAAAAGACTCTGAGTCTCGCCTGTCTTTACACTCGGACCTCGT CGACATGCATT
hTAF8_YDG30_011	ACGGGCTCACGGTACGTCGGAGTTTTGTTACACTCGGACCTCGT CGACATGCATT
hTAF8_YDG30_012	GTAGGTGTGGGGATCAGGGAACTCAGGATTACACTCGGACCTCG TCGACATGCATT
hTAF8_YDG30_013	TGATTGGTCACCGGAGGAGCAGTGATGTTACACTCGGACCTCGT CGACATGCATT
hTAF8_YDG30_014	GGTTATCGATGATGTTCTCCTCCCCATTACACTCGGACCTCGTCG ACATGCATT
hTAF8_YDG30_015	CATCCTCTGAGACCGTTTTGCATAAGCTTACACTCGGACCTCGTC GACATGCATT
hTAF8_YDG30_016	GGAGAGTGTCCACATTGAAACCCATCTTTACACTCGGACCTCGT CGACATGCATT
hTAF8_YDG30_017	ACAAGTGTGACCACGATATCGGACAGTGTTTACACTCGGACCTC GTCGACATGCATT
hTAF8_YDG30_018	TGGGTCCTGGCTGTGTGCTCACAGTATTACACTCGGACCTCGTCG ACATGCATT
hTAF8_YDG30_019	GCTCTGCAGCATCTCTGTCAGCGTTTTTACACTCGGACCTCGTCG ACATGCATT
hTAF8_YDG30_020	ACGGATGCTTTCTCGGCACTCTCAAATTACACTCGGACCTCGTCG ACATGCATT

hTAF8_YDG30_021	TGCCTCTGTCAGCAAGGAGCTCACAATTACACTCGGACCTCGTC GACATGCATT
hTAF8_YDG30_022	AGGGTTCTCCTCCGGGCCAGATGATATTACACTCGGACCTCGTC GACATGCATT
hTAF8_YDG30_023	TATCGGCAGGGTTAGTGGACTGTTTACTTCTTACACTCGGACCTC GTCGACATGCATT
hTAF8_YDG30_024	GAAAGTGGGTGATGGGTACATAGGATCTCTTTACACTCGGACCT CGTCGACATGCATT

TAF10 probes

**Name**	**Probe Sequence (including FLAPY Sequence)**
hTAF10_YDG32_001	CCATACTCGCTGAGGGCAGGGGTCAATTACACTCGGACCTCGT CGACATGCATT
hTAF10_YDG32_002	AAGTTTATTATGAAAACAGGCTGGTGTGGGGATTACACTCGGA CCTCGTCGACATGCATT
hTAF10_YDG32_003	CCTCCATGGTTAGAGTGTACTTGCGGTCTTACACTCGGACCTCG TCGACATGCATT
hTAF10_YDG32_004	CTGCCGGAGGCCGTGCCCTTCATTTTTACACTCGGACCTCGTC GACATGCATT
hTAF10_YDG32_005	AGTGCTGTAGGGCATCATTGGCAATATCTTTACACTCGGACCTC GTCGACATGCATT
hTAF10_YDG32_006	GATGAATTTCTGGGCAGCTAAGGAGATGAGCTTACACTCGGAC CTCGTCGACATGCATT
hTAF10_YDG32_007	AATTATGCGTGGGTCTGAGGCCTCAAAGCTTACACTCGGACC TCGTCGACATGCATT
hTAF10_YDG32_008	GCACGGTTCAGGTAGTAACCAGTCACTGCTTACACTCGGACC TCGTCGACATGCATT
hTAF10_YDG32_009	CTGGGATCGTAGGCGTGTAATCTTCCAGCTTACACTCGGACCTC GTCGACATGCATT
hTAF10_YDG32_010	CATCAAGAAGTCCACCAAAGGCGTGCTGGTTACACTCGGACCT CGTCGACATGCATT
hTAF10_YDG32_011	ACCACGGGCTTCACGTCTCCGTTGGCTTACACTCGGACCTCGTC GACATGCATT
hTAF10_YDG32_012	CGCTCGGCAGTACGTAAACCCCGTTAGATTACACTCGGACCTC GTCGACATGCATT
hTAF10_YDG32_013	TGGGGACAGATAAGTACATTTAGGTTGGGTGGTTACACTCGGA CCTCGTCGACATGCATT
hTAF10_YDG32_014	CCTTGTTCTCCGCGGCGGTGCTGGAGTTACACTCGGACCTCGTC GACATGCATT

hTAF10_YDG32_015	CAGGTGAAGTAGTGCGGCTTCTTCACATTGATTACACTCGGACC TCGTCGACATGCATT

TAF6 probes

Name	Probe Sequence (including FLAPY Sequence)
TAF6_1	ATGTACTTCTGGACACTCCCAGGACCTTACACTCGGACCT CGTCGACATGCATT
TAF6_2	CCAAGGGACCCGAATTCTGCCCGATATTACACTCGGACCT CGTCGACATGCATT
TAF6_3	CACATGGTCTGCTCCAATCCGGTCAATTTACACTCGGACC TCGTCGACATGCATT
TAF6_4	TGATAACATCGTGTCCCAGCTCAGCCTTACACTCGGACCT CGTCGACATGCATT
TAF6_5	AACTGTCTGCTCACGATGCAGGTCATCTTACACTCGGACC TCGTCGACATGCATT
TAF6_6	AGCTGGAATCAGCTCATGGACGTATTTTTCTTTACACTCG GACCTCGTCGACATGCATT
TAF6_7	AGGTTGTTCTGAACCACGTTCACACGGACTTACACTCGGACCTCGTCGACA TGCATT
TAF6_8	CTGCTGCTCCACAGACAACTCGTGGATTACACTCGGACCTCGTCGACATGC ATT
TAF6_9	CTCAGATCAACCTCCTTCTCCTCATAGTTACACTCGGACCTCGTCGACATGC ATT
TAF6_10	TGAACTCCTGGGCGTGGAAGCCATAGATTACACTCGGACCTCGTCGACATG CATT
TAF6_11	GCTGGGAGCCATTGGCTTTTGGAGTCCTTACACTCGGACCTCGTCGACATG CATT
TAF6_12	TTCTCGTCCACCCAGCTCTTGGTGAATTACACTCGGACCTCGTCGACATGCA TT
TAF6_13	TCTTGGTGATCCGGGACTGGATGTTGTTAGTTTACACTCGGACCTCGTCGAC ATGCATT
TAF6_14	AGTGATTGTCCACATCTGGTCGCAGGCTTACACTCGGACCTCGTCGACATG CATT
TAF6_15	ATAGAGCGTGGGGTTGTCCATCAGCGCTTACACTCGGACCTCGTCGACATG CATT
TAF6_16	CCTCCGAGATAAAGGTACTGAACCGTGGCTTACACTCGGACCTCGTCGACA TGCATT
TAF6_17	CATCTGATACAGTCCAGGGTCCGTGGCTTACACTCGGACCTCGTCGACATG CATT

TAF6_18	GGCTTGGCTGACTTCAGGGGTTCTGTGTTACACTCGGACCTCGTCGACATG CATT
TAF6_19	TTCAGCCTTCTGTTGCTCTTTGGGAGCTTACACTCGGACCTCGTCGACATGC ATT
TAF6_20	CTCGATGCTCAGCCAATGAGCTTTGAGGTTACACTCGGACCTCGTCGACAT GCATT
TAF6_21	GGCTCGACATTCTTTAGCTTCAAGGCGTATTACACTCGGACCTCGTCGACA TGCATT
TAF6_22	CAATGTCACTGGTGGTGAGCTTCTGCCTTACACTCGGACCTCGTCGACATG CATT
TAF6_23	CTGTGCGATCTCTTTGATGCGGTAGCTGTTACACTCGGACCTCGTCGACATG CATT
TAF6_24	CTCATCCGTTAGCAGCTGGCAGGTCTTTACACTCGGACCTCGTCGACATGC ATT

TAF9 probes

Name	Probe Sequence (including FLAPY Sequence)
TAF9_1	CCAACACTTAACCGCGGGACTGTTATTCTTTACACTCGGACCTCGTCGACA TGCATT
TAF9_2	CGGCACTGGATTGCCAATCGCACATCTTACACTCGGACCTCGTCGACATGC ATT
TAF9_3	CTGCATCAACAGTAGCTTTCTTAGCATGGTTACACTCGGACCTCGTCGACA TGCATT
TAF9_4	TTTGCATCATCTAGAATTGTGGTCACATATCGTTACACTCGGACCTCGTCG ACATGCATT
TAF9_5	ACTCCATGATATCCGATGATCAGACTTCTTTACACTCGGACCTCGTCGACA TGCATT
TAF9_6	TGAGGGACATGGGAGTCCCTACTTTAGTTGTTACACTCGGACCTCGTCGAC ATGCATT
TAF9_7	ACAGACATGGTCTGTGGGGTTGGTGTGCTTACACTCGGACCTCGTCGACAT GCATT
TAF9_8	AGTGTGGGAGTACTTGGTCTGCTAGTAACTGTTACACTCGGACCTCGTCGA CATGCATT
TAF9_9	GTTGATGCCTTTTTCTGTAAAGATTTCAGCCTTTACACTCGGACCTCGTCGA CATGCATT
TAF9_10	AAGGGGTTTGATTTCTTTGCCTTGCAATATCTTTACACTCGGACCTCGTCG ACATGCATT
TAF9_11	ATTTGTGCCATCATCTGTGCATCTTTCGTTACACTCGGACCTCGTCGACATG CATT

TAF9_12	ATGCTCTTGGGAGAAGCCGTCTTGCCTTACACTCGGACCTCGTCGACATGCATT
TAF9_13	GATTTTGACGCAAGTTCTTTGCCTAGTGTGGTTACACTCGGACCTCGTCGACATGCATT
TAF9_14	AGACCAAGTATACATGTTACATTCAGCAAGGCTTACACTCGGACCTCGTCGACATGCATT
TAF9_15	TGGATTAATCAGAACATTCTGAACTGCTGAGGTTACACTCGGACCTCGTCGACATGCATT
TAF9_16	GCAGGAATTGAAGCTTTTACAGCTGGAGACTTTACACTCGGACCTCGTCGACATGCATT
TAF9_17	GAAGTAGGCATCTGTACTGTAAACCTTTGACCTTACACTCGGACCTCGTCGACATGCATT
TAF9_18	CAACCTAGGACCTGAATATGGCTTGATCAATTTACACTCGGACCTCGTCGACATGCATT
TAF9_19	AGGCAAACTCCAACATCTGATTTATAACTCTTTTACACTCGGACCTCGTCGACATGCATT
TAF9_20	AGCTAAATCACCCACATTAATGTATTTCAGTCTTACACTCGGACCTCGTCGACATGCATT
TAF9_21	ACTGTTTGTGAAATACTACTTATCACACTGCGTTACACTCGGACCTCGTCGACATGCATT
TAF9_22	CCACCTTCTGCCTTTCCTTTTATTTTTGAGATTACACTCGGACCTCGTCGACATGCATT

CTNNB1 probes[10]

**Name**	**Probe Sequence (including FLAPY Sequence)**
CTNNB1_P01	CTCATGTTCCATCATGGGGTCCATACCTTACACTCGGACCTCGTCGACATGCATT
CTNNB1_P02	GCATCCTGGCCATATCCACCAGAGTGTTACACTCGGACCTCGTCGACATGCATT
CTNNB1_P03	TGTTCTGAAGAGAGAGCTGGTCAGCTCAACTTTACACTCGGACCTCGTCGACATGCATT
CTNNB1_P04	GCCGTTTCTTGTAATCTTGTGGCTTGTCCTTTACACTCGGACCTCGTCGACATGCATT
CTNNB1_P05	AGCTGTGGCTCCCTCAGCTTCAATAGTTACACTCGGACCTCGTCGACATGCATT
CTNNB1_P06	TGCAGCTTCCTTGTCCTGAGCAAGTTCATTACACTCGGACCTCGTCGACATGCATT

CTNNB1_ P07	GAGCTAGGATGTGAAGGGCTCCGGTACAACTTACACTCGGACCTCG TCGACATGCATT
CTNNB1_ P08	AAATTGCTGCTGTGTCCCACCCATGGTTACACTCGGACCTCGTCGAC ATGCATT
CTNNB1_ P09	GGCCAGTGGGATGGTGGGTGTAAGAGCTTACACTCGGACCTCGTCG ACATGCATT
CTNNB1_ P10	TGGGCCATCTCTGCTTCTTGGTGTCGTTACACTCGGACCTCGTCGAC ATGCATT
CTNNB1_ P11	TGATGTCTTCCCTGTCACCAGCCCGATTACACTCGGACCTCGTCGAC ATGCATT
CTNNB1_ P12	GTCCCAAGGAGACCTTCCATCCCTTCTTACACTCGGACCTCGTCGAC ATGCATT
CTNNB1_ P13	AGCACCTTCAGCACTCTGCTTGTGGTTTACACTCGGACCTCGTCGAC ATGCATT
CTNNB1_ P14	ACCACTAGCCAGTATGATGAGCTTGCTTTTTACACTCGGACCTCGTC GACATGCATT
CTNNB1_ P15	TTGTTTTGTTGAGCAAGGCAACCATTTTCTGCTTACACTCGGACCTC GTCGACATGCATT
CTNNB1_ P16	TGGGAAAGGTTATGCAAGGTCCCAGCGGTATTACACTCGGACCTCG TCGACATGCATT
CTNNB1_ P17	ATAGCGTGTCTGGAAGCTTCCTTTTTAGAAAGTTACACTCGGACCTC GTCGACATGCATT
CTNNB1_ P18	TGGTCCTCGTCATTTAGCAGTTTTGTCAGTTCTTACACTCGGACCTCG TCGACATGCATT
CTNNB1_ P19	ATTGCACGTGTGGCAAGTTCTGCATCATCTTACACTCGGACCTCGTC GACATGCATT
CTNNB1_ P20	ATGGTTCAGCCAAACGCTGGACATTAGTGGTTACACTCGGACCTCGT CGACATGCATT
CTNNB1_ P21	GTCCATCAATATCAGCTACTTGTTCTTGAGTGTTACACTCGGACCTC GTCGACATGCATT
CTNNB1_ P22	CTTGGGAGGTATCCACATCCTCTTCCTTTACACTCGGACCTCGTCGA CATGCATT
CTNNB1_ P23	ATTGCCTTTACCACTCAGAGAAGGAGCTGTTTACACTCGGACCTCGT CGACATGCATT
CTNNB1_ P24	GTGGCACCAGAATGGATTCCAGAGTCCAGTTACACTCGGACCTCGTC GACATGCATT

Supplementary References

1.Bardot, P., et al., The TAF10-containing TFIID and SAGA transcriptional complexes are dispensable for early somitogenesis in the mouse embryo. *Development* **2017,** *144* (20), 3808-3818.

2.Wieczorek, E., et al., Function of TAF(II)-containing complex without TBP in transcription by RNA polymerase II. *Nature* **1998,** *393* (6681), 187-91.

3.Mohan, W. S., Jr., et al., TAF10 (TAF(II)30) is necessary for TFIID stability and early embryogenesis in mice. *Mol Cell Biol* **2003,** *23* (12), 4307-18.

4.Brou, C., et al., Distinct TFIID complexes mediate the effect of different transcriptional activators. *Embo j* **1993,** *12* (2), 489-99.

5.Choukrallah, M. A., et al., Interconversion between active and inactive TATA-binding protein transcription complexes in the mouse genome. *Nucleic Acids Res* **2012,** *40* (4), 1446-59.

6.Zhao, Y., et al., A TFTC/STAGA module mediates histone H2A and H2B deubiquitination, coactivates nuclear receptors, and counteracts heterochromatin silencing. *Mol Cell* **2008,** *29* (1), 92-101.

7.Nagy, Z., et al., The metazoan ATAC and SAGA coactivator HAT complexes regulate different sets of inducible target genes. *Cellular and molecular life sciences : CMLS* **2010,** *67* (4), 611-28.

8.Jacq, X., et al., Human TAFII30 is present in a distinct TFIID complex and is required for transcriptional activation by the estrogen receptor. *Cell* **1994,** *79* (1), 107-17.

9.May, M., et al., Human TAF(II28) promotes transcriptional stimulation by activation function 2 of the retinoid X receptors. *Embo j* **1996,** *15* (12), 3093-104.

10.Tsanov, N., et al., smiFISH and FISH-quant - a flexible single RNA detection approach with super-resolution capability. *Nucleic Acids Res* **2016,** *44* (22), e165.
