# Supplementary material for: Co-translational assembly of mammalian nuclear multisubunit complexes
Source: Nat Commun. 2019 Apr 15;10:1740. doi: 10.1038/s41467-019-09749-y (PMC6465333; doi:10.1038/s41467-019-09749-y)
Supplement: Supplementary file 3 — Reporting Summary [file 41467_2019_9749_MOESM3_ESM.pdf]

nature research

Corresponding author(s): Laszlo Tora

Last updated by author(s): Mar 22, 2019

Reporting Summary

Nature Research wishes to improve the reproducibility of the work that we publish. This form provides structure for consistency and transparency in reporting. For further information on Nature Research policies, see Authors & Referees and the Editorial Policy Checklist.

Statistics

For all statistical analyses, confirm that the following items are present in the figure legend, table legend, main text, or Methods section.

n/a	Confirmed	
☐	☒	The exact sample size (n) for each experimental group/condition, given as a discrete number and unit of measurement
☐	☒	A statement on whether measurements were taken from distinct samples or whether the same sample was measured repeatedly
☐	☒	The statistical test(s) used AND whether they are one- or two-sided<br>*Only common tests should be described solely by name; describe more complex techniques in the Methods section.*
☒	☐	A description of all covariates tested
☒	☐	A description of any assumptions or corrections, such as tests of normality and adjustment for multiple comparisons
☐	☒	A full description of the statistical parameters including central tendency (e.g. means) or other basic estimates (e.g. regression coefficient) AND variation (e.g. standard deviation) or associated estimates of uncertainty (e.g. confidence intervals)
☐	☒	For null hypothesis testing, the test statistic (e.g. F, t, r) with confidence intervals, effect sizes, degrees of freedom and P value noted<br>*Give P values as exact values whenever suitable.*
☒	☐	For Bayesian analysis, information on the choice of priors and Markov chain Monte Carlo settings
☒	☐	For hierarchical and complex designs, identification of the appropriate level for tests and full reporting of outcomes
☒	☐	Estimates of effect sizes (e.g. Cohen's d, Pearson's r), indicating how they were calculated

Our web collection on statistics for biologists contains articles on many of the points above.

Software and code

Policy information about availability of computer code

Data collection	Imaging of smiFISH and IF-smiFISH samples was performed on an SP8UV microscope (Leica) equipped with a 633-nm HeNe laser, a 561-nm DPSS laser, a 488-nm argon laser and a 405-nm laser diode. A 63x oil immersion objective (NA 1.4) was used and images were taken by using the hybrid detector photon-counting mode. The laser power for all acquisitions and laser lines was set to 10%. All images acquired have a bit depth of 8 bit and a pixel resolution of 70 nm. The z-stacks were taken with a z-spacing of 300 nm for a total of 4-6 µm. Image processing was performed using the Fiji/Image J software.<br>The microarray results reported in this paper are available in the Gene Expression Omnibus (GEO) under accession number GSE106299. RIP-qPCR calculations were done and Figure panels were prepared using R (RStudio version 1.1.456 and R version 3.5.1).
Data analysis	For both smiFISH and IF-smiFISH, mRNAs were detected with FISH-quant (Tsanov et al, 2016). Matlab script for IF-smiFISH co-localization analysis is available upon request. We implemented a user interface for dual color smiFISH analysis tool (FQ_DualColor), which is distributed together with a dedicated user manual with FISH-quant: https://bitbucket.org/muellerflorian/fish_quant.

For manuscripts utilizing custom algorithms or software that are central to the research but not yet described in published literature, software must be made available to editors/reviewers. We strongly encourage code deposition in a community repository (e.g. GitHub). See the Nature Research guidelines for submitting code & software for further information.

Data

Policy information about availability of data

All manuscripts must include a data availability statement. This statement should provide the following information, where applicable:

- Accession codes, unique identifiers, or web links for publicly available datasets
- A list of figures that have associated raw data
- A description of any restrictions on data availability

The microarray data corresponding to Figure 1a and 7a are available in the Gene Expression Omnibus (GEO) under accession number GSE106299. The source data corresponding to Figs. 1d-e, 2a-d, 3a-b, 4a-d, 6a-d, 7b-f, 8a-c and Supplementary Figs. 1, 2a-d, 3a-b, 5a-d, 7a-c are provided as a Source Data file. Raw image files

(approximately 800) and their corresponding analyses are available upon request. The Matlab script for Kamenova_NatComm__rna_protein_coloc.m concerning the RNA co-localization and IF-smiFISH analyses are available on the FISH-quant repository https://bitbucket.org/muellerflorian/fish_quant.

Field-specific reporting

Please select the one below that is the best fit for your research. If you are not sure, read the appropriate sections before making your selection.

☒ Life sciences ☐ Behavioural & social sciences ☐ Ecological, evolutionary & environmental sciences

For a reference copy of the document with all sections, see nature.com/documents/nr-reporting-summary-flat.pdf

Life sciences study design

All studies must disclose on these points even when the disclosure is negative.

Sample size	All biochemical experiments were performed in at least 2 biological replicates except Figure 1d and 7b which was done in triplicates. Every conclusion drawn in the study was validated many times by several alternate approaches.
Data exclusions	No data was excluded.
Replication	RIP-qPCR data reported in all the figures were calculated from 2 biological replicates except Figure 1d and Figure 7b where the data were reported from 3 biological replicates. IF-smiFISH experiments were repeated at least 4 times. Dual colour smiFISH experiments were done in 2 biological replicates. The experiments provided in the manuscript were all reproducible.
Randomization	*Describe how samples/organisms/participants were allocated into experimental groups. If allocation was not random, describe how covariates were controlled OR if this is not relevant to your study, explain why.*
Blinding	*Describe whether the investigators were blinded to group allocation during data collection and/or analysis. If blinding was not possible, describe why OR explain why blinding was not relevant to your study.*

Reporting for specific materials, systems and methods

We require information from authors about some types of materials, experimental systems and methods used in many studies. Here, indicate whether each material, system or method listed is relevant to your study. If you are not sure if a list item applies to your research, read the appropriate section before selecting a response.

Materials & experimental systems

n/a	Involved in the study	
☐	☒	Antibodies
☐	☒	Eukaryotic cell lines
☒	☐	Palaeontology
☒	☐	Animals and other organisms
☒	☐	Human research participants
☒	☐	Clinical data

Methods

n/a	Involved in the study	
☒	☐	ChIP-seq
☒	☐	Flow cytometry
☒	☐	MRI-based neuroimaging

Antibodies

Antibodies used	The catalogue numbers for commercial antibodies are : HA, 3F10 (Sigma cat #: 11867423001), anti-FLAG M2 (Sigma, F1804), GANP (ab113295), SUPT7L (Bethyl, A302-803A). All the other antibodies used are published. A complete list of antibodies used in this study and their dilution used are provided with their reference in our Supplementary Table 2.
Validation	Validation statements of antibodies : 23TA-1H8 (Wieczorek et al, 1998), 6TA-2B11 (Mohan et al, MCB, 2003), 3TF1-3G3 (Brou et al, EMBO J, 1993), 3F10 (https://www.sigmaaldrich.com/content/dam/sigma-aldrich/docs/Roche/Bulletin/1/roahahabul.pdf), anti-FLAG M2 (https://www.sigmaaldrich.com/content/dam/sigma-aldrich/docs/Sigma/Bulletin/f1804bul.pdf), #2440 (Choukrallah et al, 2011), #2325 (Zhao et al, 2008), hGANP (https://www.abcam.com/ganp-antibody-ab113295.html#top-0), SUPT7L (https://www.bethyl.com/product/A302-803A/SUPT7L+Antibody), #3478 (Bardot et al, 2017), 15-TF2-1D10 (Nagy et al., 2010).

Eukaryotic cell lines

Policy information about cell lines

Cell line source(s)	All the cell lines except GFP-TAF1 used in the study were obtained from IGBMC cell culture facility. GFP-TAF1 expressing HeLa cell line was obtained from H.T. Marc Timmers' lab (DKTK, Freiburg, Germany).
Authentication	To ensure reliable, reproducible results, the HeLa cell line used were authenticated and quality-tested by ATCC. The E14 mouse embryonic stem cells (ES Parental cell line E14Tg2a.4) were obtained from Mutant Mouse Resource and Research Center (MMRRC) (Citation ID:RRID:MMRRC_015890-UCD).
Mycoplasma contamination	All cell lines used in the study were tested for Mycoplasma contamination by the IGBMC Cell Culture Facility and were negative.
Commonly misidentified lines (See ICLAC register)	*Name any commonly misidentified cell lines used in the study and provide a rationale for their use.*
